# Air exposure and cell differentiation are essential for investigation of SARS-CoV-2 entry genes in human primary airway epithelial cells *in vitro*

**DOI:** 10.3389/fmed.2022.897695

**Published:** 2022-09-06

**Authors:** Brigitte Kasper, Xiaoyang Yue, Torsten Goldmann, Askin Gülsen, Christian Kugler, Xinhua Yu, Frank Petersen

**Affiliations:** ^1^Priority Area Chronic Lung Diseases, Research Center Borstel, Member of the German Center for Lung Research (DZL), Borstel, Germany; ^2^Pathology, Research Center Borstel, Member of the German Center for Lung Research (DZL), Borstel, Germany; ^3^Department of Surgery, LungenClinicGrosshansdorf, Grosshansdorf, Germany

**Keywords:** primary human airway epithelial cells, SARS-CoV-2 entry genes, submerged culture, ALI culture, COVID-19

## Abstract

**Background:**

*In-vitro* models of differentiated primary human airway epithelial cells are a valuable tool to study severe acute respiratory syndrome coronavirus 2 (SARS-CoV-2) infection. Through the use of these models, it has been shown that the expression of SARS-CoV-2 entry genes in human airway epithelia is influenced by various factors such as age, sex, smoking status, and pathogenic conditions. In this study, we aimed to determine the effects of cell culture conditions and donor demographic and clinical characteristics on the expression of SARS-CoV-2 entry genes including angiotensin converting enzyme 2 (ACE2), transmembrane serine protease 2 (TMPRSS2), cathepsin L (CTSL), and tyrosine protein kinase receptor UFO (AXL) in primary airway epithelial cells.

**Methods:**

Eleven lung cancer patients with or without chronic obstructive pulmonary disease (COPD) or asthma were recruited. Human bronchial epithelial cells (HBEC) or small airway epithelial cells (SAEC) isolated from submerged or air-liquid interface (ALI) cultures were analyzed by quantitative real-time PCR. We also tested for correlations with clinical data.

**Results:**

In ALI cultures, the expression of AXL was significantly higher in HBEC than in SAEC. In addition, the expression of ACE2, TMPRSS2, and CTSL was significantly increased in both HBEC and SAEC differentiated under ALI conditions compared with the submerged culture. Negligible association was found between the expression of SARS-CoV-2 entry genes in SAEC and the age, sex, smoking status, and complication of COPD, asthma or hypertension of the cell donors.

**Conclusion:**

These results demonstrate that the expression of SARS-CoV-2 entry genes in differentiated primary airway epithelial cells *in-vitro* is much more influenced by individual culture conditions than by specific characteristics of individual donors.

## Introduction

The interaction between the virus spike (S) protein and entry receptors on human respiratory epithelial cells is a critical step for infection with severe acute respiratory syndrome coronavirus 2 (SARS-CoV-2), the causative agent of 2019 coronavirus disease (COVID-19) (1). As the major cell entry receptor for SARS-CoV-2, angiotensin converting enzyme 2 (ACE2) recognizes the S1 receptor binding domain and mediates virus entry *via* two major pathways, cathepsin L-dependent endocytosis and transmembrane serine protease 2 (TMPRSS2)-dependent membrane fusion (1–3). Besides ACE2, the tyrosine protein kinase receptor UFO (AXL) was recently reported to be another entry receptor for SARS-CoV-2 (4). In contrast to ACE2, AXL promotes viral entry by interacting with the N-terminal domain of the SARS-CoV-2 S protein (4).

*In-vitro* modeling of SARS-CoV-2 infection in primary human airway epithelial cells is a valuable tool for understanding the molecular mechanisms underlying viral infection and for the search for potential COVID-19 drugs (5). In these *in-vitro* systems, primary human airway epithelial cells isolated from surgical resections of patients with various diseases are propagated in different cell culture systems, including submerged, air-liquid interface (ALI), and 3-dimensional cell cultures (3D; spheroids or bronchospheres) (6). Furthermore, age, sex, smoking status, and airway clinical complications have been reported to be associated with ACE2 expression in human airway epithelium (7–9). In addition, epithelial cells isolated from different parts of the airways, such as the nasal passages, bronchi, and small airways, also have different expression levels of ACE2 (10–12), which may further increase the complexity of modeling these differences with *in-vitro* systems.

In this study, we aimed to investigate the effects of different types of airway epithelial cells and cell culture conditions, as well as age, sex, smoking status, and clinical characteristics of the subjects on the expression of SARS-CoV-2 entry genes in differentiated cells *in-vitro*. For this purpose, human bronchial epithelial cells (HBEC) and small airway epithelial cells (SAEC) were isolated from surgical biopsies of the lungs of 11 patients with lung cancer and cultured *in vitro*. Our results show that the expression of SARS-CoV-2 entry genes was strongly influenced by cell culture systems, whereas it was not significantly related to patient age, sex, and smoking status or pulmonary co-morbidities such as chronic obstructive pulmonary disease (COPD) and asthma.

## Materials and methods

### Materials

Collagen I from human skin, Pronase, DNase I, SBTI, accutase, and IgG from human serum were purchased from Sigma-Aldrich; PneumaCult™-EX PLUS, PneumaCult™-ALI were from STEMCELL Technologies; EASYstrainer Cell Sieves (100 and 40 μm), transwell inserts and 96 well plates with cell-repellent surface were from Greiner Bio-One; High Pure RNA Isolation kit, Transcriptor First Strand cDNA synthesis kit, SYBR Green PCR Master Mix were from Roche Diagnostics.

Antibodies for flow cytometry staining were ACE2-AlexaFluor 647 (clone E-11), AXL-AlexaFluor647 (clone B-2), acetylated α-Tubulin-AlexaFluor 488 (clone 6-11B-1), MUC5AC-AlexaFluor 488 (clone 45M1), CC10-AlexaFluor 488 (clone E-11), Cytokeratin 5-AlexaFluor 488 (clone RCK103) were purchased from Santa Cruz Biotechnology; Mouse IgG1-AlexaFluor 488, Mouse IgG1-AlexaFluor 647 were from BioLegend, Mouse IgG2b-FITC was from DAKO; and Live/Dead™ Fixable Blue Dead Cell Stain was from Invitrogen.

### Patients

Lung tissue and/or bronchus from 11 patients were provided by the Pathology Department (Research Center Borstel, Germany). All patients underwent lung resection and were characterized as lung cancer stages ranging from IA to IIB. These were samples from residual material without a declaration of consent (AZ 12-220) with defined accompanying data. Here, the regulations of the Bio Material Bank Nord are applied (AZ 14-225). The study was conducted in accordance with the Declaration of Helsinki. The use of biomaterial and data for this study was approved by the local ethics committee of the University Lübeck (AZ 17-131). Sex, age, and smoking behavior, and additional information for every individual are provided in Table 1 and Supplementary Table S1.

**Table 1:** Demographic and clinical features of patients recruited for the isolation of airway epithelial cells.

	**Lung cancer**	**Lung cancer + asthma**	**Lung cancer + COPD**
Number of patients (total = 11)	4	4	3
Male/female	3/1	2/2	2/1
Age [years] (range)	74.0 (68–83)	69.3 (57–81)	71.7 (70–75)
Smoking status (current/ex-smoker)	3/1	2/2	0/3
Hypertension, *n* (4/11)	2	2	0

### Isolation and submerged culture of primary human airway epithelial cells

Tissue was assembled during routine surgical intervention from lung cancer patients. Since it has been reported that there are significant difference in expression of SARS-CoV-2 entry genes between tumor and the adjacent tumor-free tissue (13), epithelial cells were isolated from tumor free tissues. Lung or bronchial tissue was placed in a 100 mm culture dish and rinsed in D-PBS. Tissue was cut in smaller pieces, excess surrounding tissue was dissected out, and bronchial tissue separated. A scalpel was used to cut open the bronchial pieces, and cut the tissue into 2–3 mm pieces, for better exposure to enzyme solution. Bronchial tissue was then transferred to a tube containing 20 ml of RPMI1640, supplemented with 2 mM L-glutamin, 1.4 mg/ml pronase, 0.1 mg/ml DNAse I and placed at 4–8°C for overnight incubation. The next day, FCS was added to 10% to stop the action of the protease, and the tube was inverted several times to mix well and dislodge cells. Bronchial tissue was discarded, and the cell-containing supernatant was transferred through EASYstrainer Cell Sieves (100 and 40 μm) to new tubes and centrifuged 10 min at 600 × g. Epithelial cells were then collected into a new tube and centrifuged after which pellet was resuspended in RPMI1640 to rinse cells and allow them to be counted. Cells were centrifuged again and the pellet was resuspended in expansion medium (PneumaCult EX PLUS™ Complete Medium), transferred to collagen I-coated (3.45 μg/cm^2^) 100 mm culture plate (150.000–300.000 cells per culture plate), and incubated at 37°C, 5% CO_2_. Medium was changed every 3–4 days until cells reached desired confluency. Cells isolated from lung tissue (secondary/tertiary bronchi and bronchioles) were named small airway epithelial cells (SAEC) and cells isolated from main (primary) bronchus were named human bronchial epithelial cells (HBEC) (Supplementary Figure S1). All cells could be expanded to passage 8–11. Most of the experiments were done with cells from passage 3–6.

### Air-liquid interface (ALI) culture of primary human airway epithelial cells

Primary human airway epithelial cells were expanded and differentiated at ALI *in-vitr*o following the protocol given by STEMCELL Technologies. Cells were expanded on collagen I-coated (3.45 μg/cm^2^) dishes in expansion medium, and harvested by trypsinization with SBTI neutralization. The apical chambers of 6.5 mm transwell inserts were coated with 5 μg/cm^2^ collagen I, and cells were seeded at 3 × 10^5^ cells/insert in expansion medium, and the basolateral chambers received 500 μL of expansion medium alone. Once the epithelial cells reached confluence, the apical growth media was removed, and the basolateral medium was replaced with PneumaCult™-ALI Maintenance Medium (ALI-MM), initiating day 0 of the ALI mucociliary culture system. Epithelial cultures were allowed to differentiate at ALI *in-vitro* for 14–21 days at 37°C, 5% CO_2_. In the basolateral chambers ALI-MM was changed every other day. Differentiation of the cells was confirmed by immunohistochemistry (Supplementary Figure S2).

### 3D culture of primary human airway epithelial cells

For the generation of spheroids 2–3 × 10^6^ cells were resuspended in 10 ml ALI-MM and seeded onto 96 well plates with cell-repellent surface (100 μl cells/well). Plates were centrifuged for 1 min at 450 × g and incubated at 37°C, 5% CO_2_ for 4–7 days. The spheroids were featured by ciliated cells on the apical, outer side (Supplementary Figure S3).

### Detection of ACE2 and AXL by flow cytometry

For flow cytometry ALI or 3D cultured cells were used. ALI cultured cells were detached from transwell membranes using accutase, and 3D cultured cells were carefully resuspended. The surface markers of cells were analyzed for the presence of ACE2, AXL, and acetylated α-Tubulin using AlexaFluor 488- or AlexaFluor 647 conjugated monoclonal antibodies (all diluted 1/100 in D-PBS/Live-Dead stain (1:1000), supplemented with 60 μg/ml human IgG (Sigma Aldrich) at 8°C for 30 min. After that, cells were washed in FACS buffer (D-PBS/0.1% BSA), fixed in 4% PFA at room temperature (RT) for 15 min and permeabilized in 0.2% Triton X-100 at RT for 20 min. Intracellular markers MUC5AC, CC10 and cytokeratin 5 were analyzed using AlexaFluor 488 conjugated monoclonal antibodies (all diluted 1/100 in FACS buffer, supplemented with 60 μg/ml human IgG) at 8°C for overnight. The cells were washed and analyzed by flow cytometry (LSRII, BD Biosciences), and post-acquisition analysis was carried out using FCSExpress 7 (De Novo Software).

### RNA extraction, cDNA synthesis and quantitative PCR

RNA was extracted from cell lysates of primary airway epithelial cells (either expanded in submerged cultures or differentiated in ALI cultures), by using the High Pure RNA Isolation kit, and reverse transcription of 0.5–1 μg of total RNA was performed using the Transcriptor First Strand cDNA synthesis kit. Amplifications of target and HPRT genes were performed using SYBR Green PCR Master Mix, following the manufacturer's instructions, in a LightCycler 480 System (Roche Diagnostics). Data analyses were done using the LightCycler 480 relative quantification software with data normalized to the mRNA level of HPRT housekeeping gene. The sequences of the primer used were synthetized by Metabion and include: *ACE2* forward: AACTACCCGGAGGGCATAG and reverse: CTGGGATGTCCGGTCATATT; *AXL* forward: AACCAGGACGACTCCATCC and reverse: AGCTCTGACCTCGTGCAGAT; *CTSL* forward: GGGAGGGCAGTTGAGGAC and reverse: GCAAGGATGAGTGTAGGATTCA; *TMPRSS2* forward: CGCTGGCCTACTCTGGAA and reverse: CTGAGGAGTCGCACTCTATCC; *HPRT* forward: TGACCTTGATTTATTTTGCATACC and reverse: CGAGCAAGACGTTCAGTCCT.

### Statistical analysis

For all statistical analyses, the GraphPad Prism 5 software package was used (GraphPad Software). For quantitative data, statistical significance between groups was determined by paired *t* test, unpaired *t* test, one-way ANOVA followed by Tukey's Multiple Comparison Test, or Kruskal-Wallis test followed by Dunn's Multiple Comparison Test. Pearson's correlation was used to measures the statistical relationship between two continuous variables. For patients whose SAEC were used in multiple cultures, mean values of gene expression levels were used for the correlation analysis. A *p* values of 0.05 or lower were considered statistically significant.

## Results

### Patients and cells

As summarized in Table 1, tumor free lung tissues from 11 patients with lung cancer was studied, including 2 with squamous cell carcinoma, 8 with adenocarcinoma and 1 with non-small cell lung cancer (NSCLC; not otherwise specified). The 11 patients were 8 former smokers and 3 current smokers, 7 men and 4 women. The mean age of the patients was 72 years and ranged from 57 to 83 years. SAEC were isolated from all 11 patients, whereas HBEC were isolated from 4 of them. The isolated primary airway epithelial cells were passaged and then cultured in submerged or ALI cultures to examine the expression of SARS-CoV-2 entry genes.

### HBEC and SAEC express comparable levels of SARS-CoV-2 entry genes with exception of AXL

**Figure 1:**
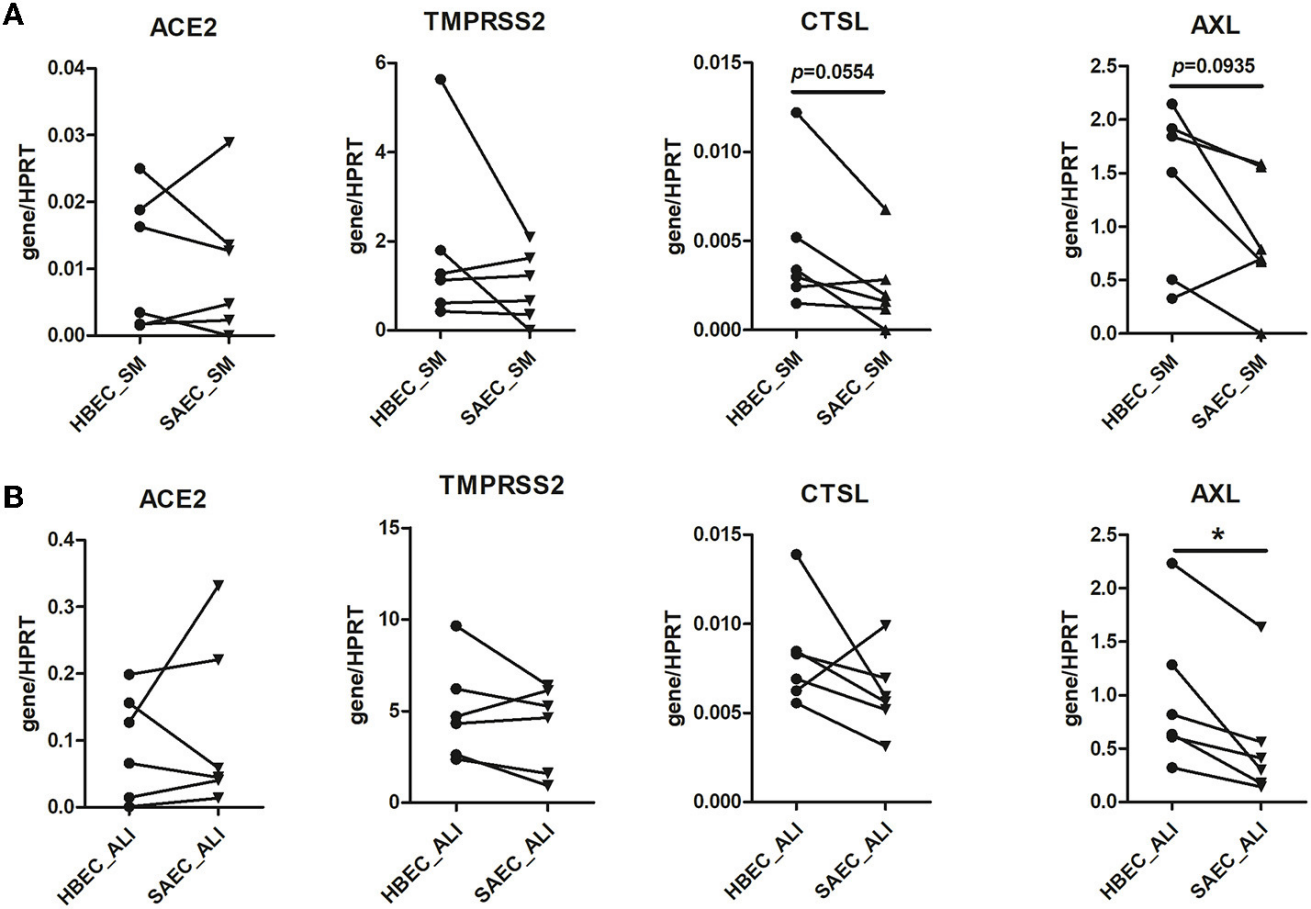
Comparison of expression of SARS-CoV-2 entry genes between human bronchial epithelial cells (HBEC) and small airway epithelial cells (SAEC). HBEC and SAEC derived from the same donors were submerged cultured (SM) **(A)** or differentiated under air-liquid interface (ALI) conditions (4 patients, 6 cultures) **(B)**. Gene expression of SARS-CoV-2 entry genes including ACE2, TMPRSS2, CTSL and AXL was determined by LightCycler PCR and normalized to HRPT expression. Comparisons on expression of the four SARS-CoV-2 genes between HBEC and SAEC from same donors are performed for cells cultured in submerged culture and ALI culture. Statistical significance was determined by paired *t* test (*n* = 6). *p < 0.05.

First, we compared HBEC and SAEC with respect to the expression of SARS-CoV-2 entry genes. HBEC and SAEC isolated from same donors were cultured under both, submerged and ALI conditions, and the expression levels of *ACE2, TMPRSS2, CSTL*, and *AX*L were determined and normalized to the expression of housekeeping gene *HPRT*. As shown in Figure 1A, none of the four genes were significant differentially expressed when compared between submerged cultured HBEC and SAEC. When cells were differentiated under in ALI conditions, no significant difference was observed between HBEC and SAEC in expression levels of *ACE2, TMPRSS2*, and *CTSL*. However, expression of *AXL* was significantly higher in HBEC than in SAEC (0.98 ± 0.28 vs. 0.54 ± 0.23, *p* = 0.0168) (Figure 1B). Therefore, with the exception of *AXL* expression under ALI culture conditions, *in-vitro* differentiated HBEC and SAEC expressed comparable levels of SARS-CoV-2 entry genes.

### SARS-CoV-2 entry gene expression is strongly associated with cell culture conditions

**Figure 2:**
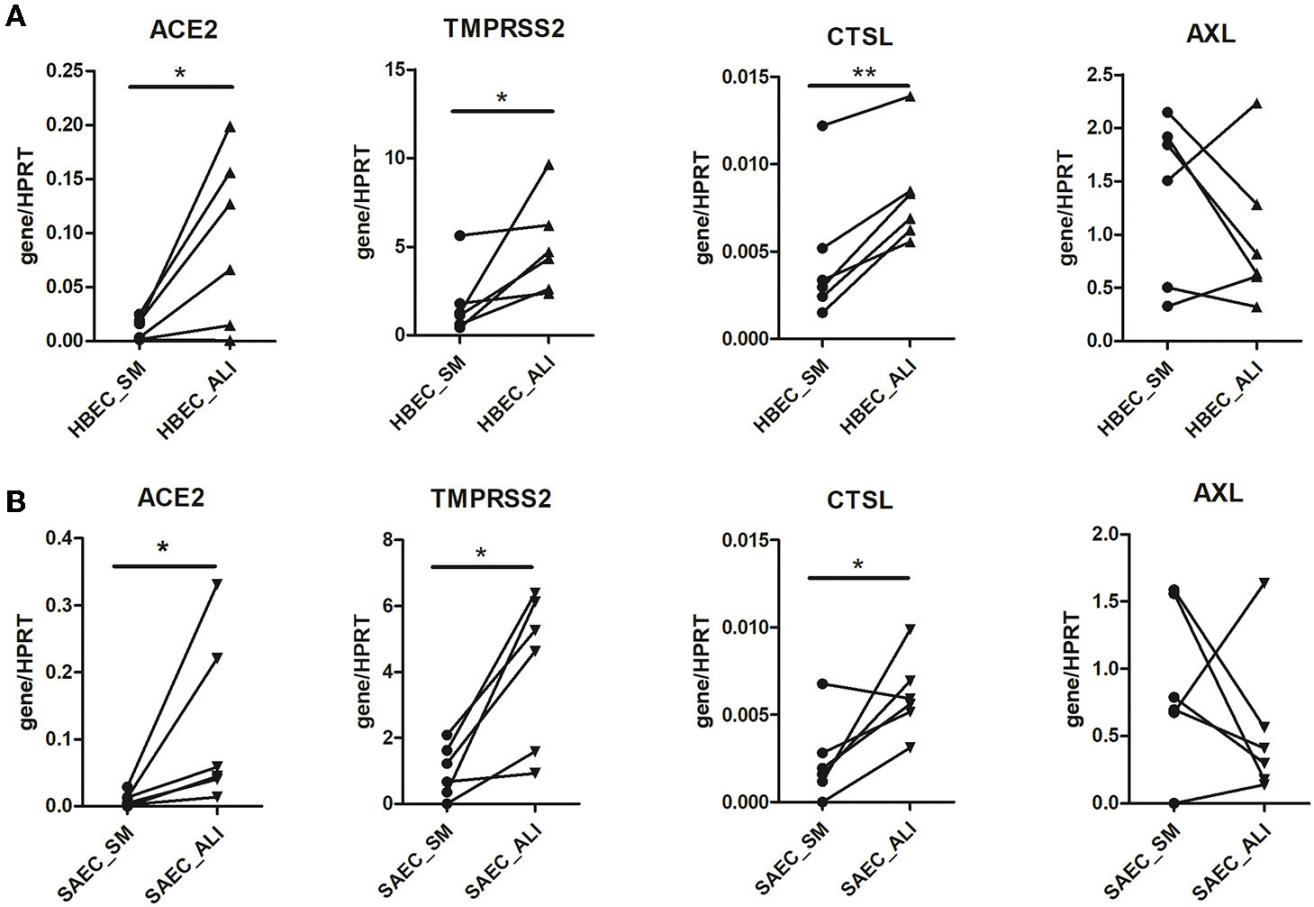
Effect of cell culture systems on the expression of SARS-CoV-2 entry genes in primary human airway epithelial cells. Human bronchial epithelial cells (HBEC) **(A)** and small airway epithelial cells (SAEC) **(B)** derived from the same donors were submerged cultured (SM) or differentiated under air-liquid interface (ALI) conditions (4 patients, 6 cultures). Gene expression of SARS-CoV-2 entry genes including ACE2, TMPRSS2, CTSL and AXL was determined by LightCycler PCR and normalized to HRPT expression. Expression of the four SARS-CoV-2 genes in HBEC and SAEC cultured in submerged culture was compared with those in ALI culture. Statistical significance was determined by paired *t* test (*n* = 6). *p < 0.05 and **p < 0.01.

The above experiments provide preliminary evidence for a possible influence of cell culture conditions on the expression of SARS-CoV-2 entry genes. Therefore, HBEC cells derived from submerged and ALI cultures were examined for the expression of SARS-CoV-2 entry genes (Figure 2A). Notably, expression of *ACE2* in HBEC differentiated under ALI condition was significantly higher than those grown in submerged culture (0.094 ± 0.032 vs. 0.011 ± 0.004, *p* = 0.0353). In addition, the expression of the other two genes involved in ACE2-mediated viral entry, *TMPRSS2* (4.99 ± 1.10 vs. 1.81 ± 0.79, *p* = 0.0313) and *CTSL* (0.0082 ± 0.0012 vs. 0.0046 ± 0.0016, *p* = 0.0018), was also significantly increased in HBEC grown under ALI conditions compared to those grown in submerged culture. Similar findings were observed in SAEC cells, where differentiation under ALI conditions significantly increased the expression of *ACE2* (0.118 ± 0.052 vs. 0.010 ± 0.004, *p* = 0.0313), *TMPRSS2* (4.16 ± 0.95 vs. 0.99 ± 0.32, *p* = 0.0121) and *CTSL* (0.0061 ± 0.0009 vs. 0.0024 ± 0.0009, *p* = 0.0349) compared with submerged culture (Figure 2B). In contrast, the expression of *AXL* was not significantly different between the two cell culture conditions in both HBEC and SAEC.

### SARS-CoV-2 entry gene expression is not associated with COPD or asthma

**Figure 3:**
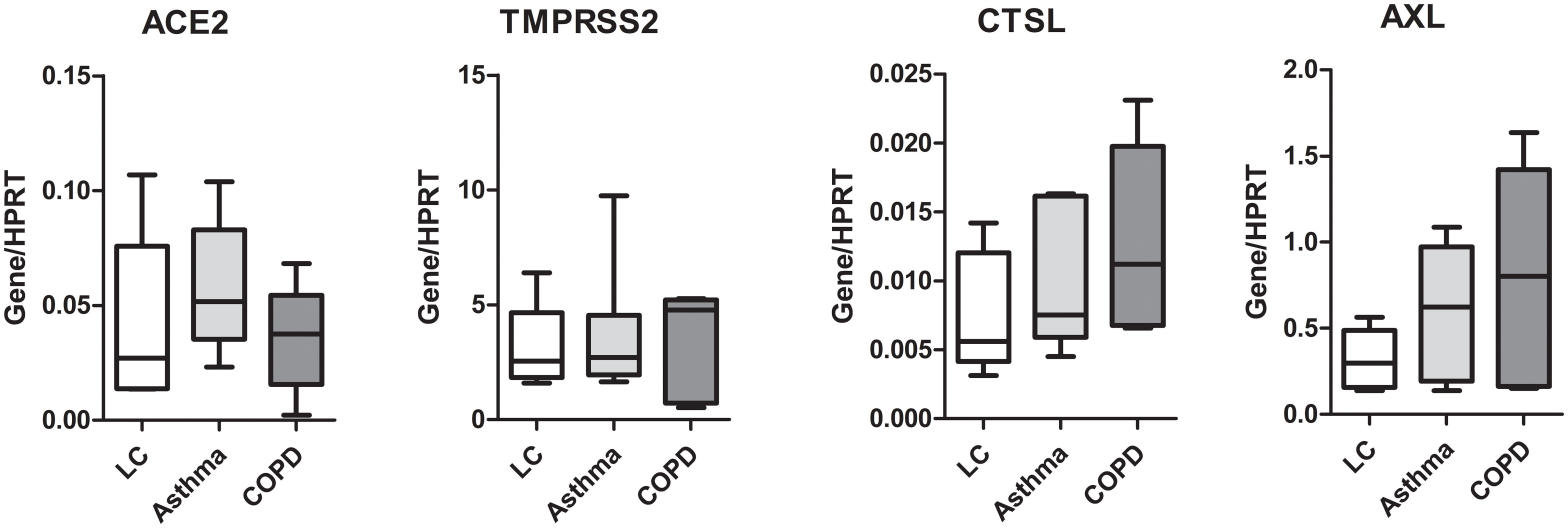
Expression of SARS-CoV-2 entry genes is not associated with asthma or COPD. SAEC isolated from patients with lung cancer (LC; 4 patients, 5 cultures), LC with asthma (4 patients, 6 cultures), and LC with COPD (3 patients, 5 cultures) were ALI cultured. Gene expression was analyzed by LightCycler PCR and normalized to HRPT expression. Data are presented as Box and Whiskers (min to max) and statistical significance was tested by one-way ANOVA followed by Tukey's Multiple Comparison Test, or Kruskal-Wallis test followed by Dunn's Multiple Comparison Test.

Because ALI culture is closer to physiological conditions than submerged culture, we focused on the expression of these genes in SAEC differentiated under the former conditions in the following experiments. Because there is evidence that respiratory diseases such as COPD and asthma may affect the expression of SARS-CoV-2 entry genes (14–16), we next examined whether SAEC derived from patients with COPD or asthma differed in gene expression from that of donors without asthma or COPD. As shown in Figure 3, the expression of *ACE2* in SAEC isolated from lung cancer patients without respiratory co-morbidities, lung cancer patients with asthma, and lung cancer patients with COPD was 0.041 ± 0.017, 0.057 ± 0.012, and 0.035 ± 0.011, respectively, with no significant differences between groups. No significant difference was also observed in the expression levels of *TMPRSS2, CTSL*, and *AXL* among SAEC isolated from the three patient groups, although there was a tendency for higher expression of *CTSL* and *AXL* in SAEC isolated from patients with co-morbidity of COPD, but this was not statistically significant.

### Minimal effect of smoking status, sex, and age of cell donors or hypertension on the expression of SARS-CoV-2 entry genes in SAEC

**Figure 4:**
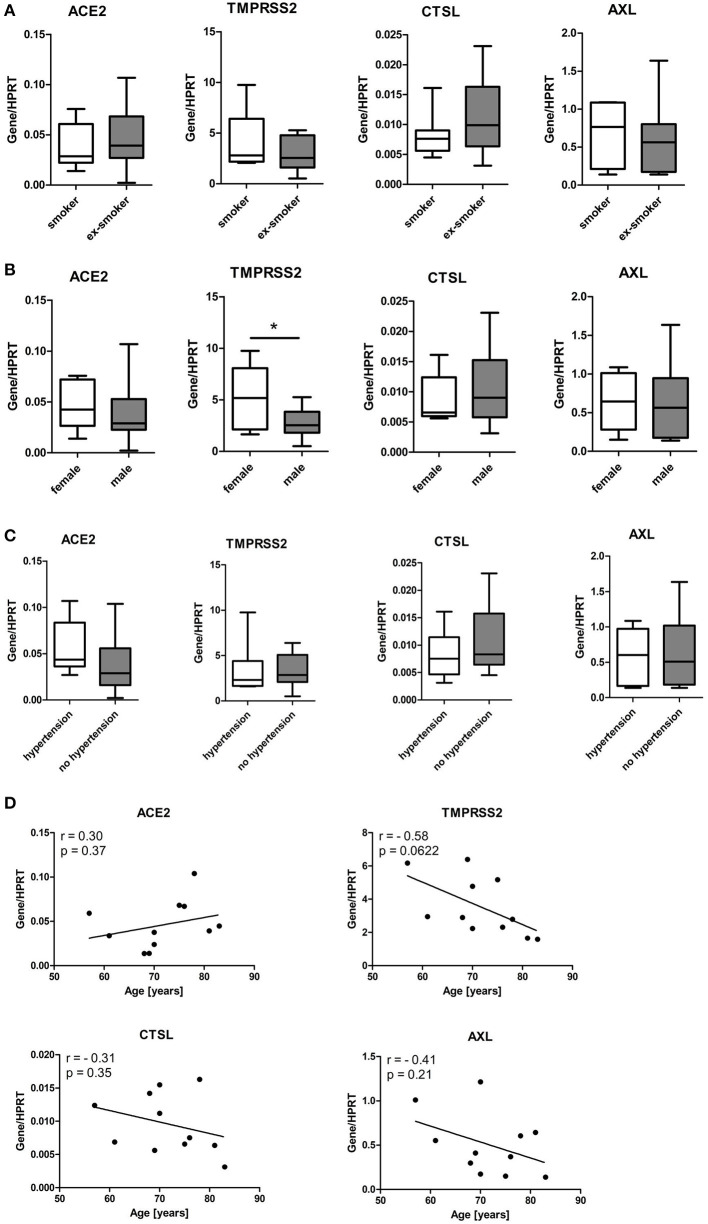
Influence of smoking status, gender, age and hypertension of patients on expression of SARS-CoV-2 entry genes in SAEC. SAEC isolated from 11 lung cancer patients were ALI cultured. Gene expression was analyzed by LightCycler PCR and normalized to HRPT expression. **(A)** Comparison of gene expression of SARS-CoV-2 entry genes between current smokers (3 patients, 7 cultures) and ex-smokers (8 patients, 11 cultures). **(B)** Comparison of gene expression of SARS-CoV-2 entry genes between males (7 patients, 13 cultures) and females (4 patients, 5 cultures). **(C)** Comparison of gene expression of SARS-CoV-2 entry genes between patients with or without hypertension (4 patients, 6 cultures or 7 patients, 12 cultures, respectively). Data were presented as Box and Whiskers (min to max) and statistical significance was determined by unpaired *t* test. *p < 0.05. **(D)** Correlation between levels of gene expression and age of 11 patients. For patients whose SAEC were used in multiple cultures, mean values of gene expression levels were used for the correlation analysis. Pearson correlation coefficient (r) and *p* values are indicated.

Given that no significant difference in expression of SARS-CoV-2 entry genes was observed between SAEC from lung cancer patients with or without respiratory complications, we next combined all samples for further analysis. We first determined the effect of smoking by comparing data sets of current smokers and ex-smokers. Expression of *ACE2* in SAEC of current smokers (0.038 ± 0.0085) was not significantly different to that of former smokers (0.046 ± 0.010). Furthermore, levels expression of *TMPRSS2, CTSL*, and *AXL* in SAEC of current smokers were also comparable to those of former smokers (Figure 4A). When these SAEC were divided into two groups according to sex of the cell donors, only a significant difference was observed between the two subgroups in the expression of *TMPRSS2*, with female SAEC showing significantly higher expression than male SAEC (Figure 4B), while comparing of the four genes in patients with or without hypertension no significant differences could be observed (Figure 4C). To evaluate the effect of age of cell donors, a correlation analysis was performed. As shown in Figure 4D, only a marginally significant inverse correlation was detected between cell donor age and *TMPRSS2* expression, with a Pearson's correlation coefficient of−0.58 (*p* = 0.0622). Also, no significant correlation was found between the age of cell donors and expression of and the expression of *ACE2, CTSL* and *AXL*.

### Surface expression of ACE2 and AXL on *in-vitro* differentiated SAEC

High expression of *ACE2, TMPRSS2* and *CTSL* in SAEC cultured under ALI condition implies that expression SARS-CoV-2 entry genes is dependent on the differentiation of SAEC. SAEC could either be differentiated under ALI conditions, or in 3D culture. Using flow cytometry, we next determined the surface protein expression of ACE2 and AXL on *in-vitro* differentiated SAEC. As shown in Supplementary Figure S4A, SAEC differentiated under ALI condition consisted of 27.00 ± 3.77% ciliated cells, 5.17 ± 1.26% goblet cells, 29.26 ± 3.28% club cells, and 24.97 ± 2.33% basal cells. Notably, majority of all four types of cell express ACE2 and AXL (Supplementary Figure S4B). Regarding the levels of surface expression of ACE2 and AXL, goblet cells showed highest expression of both ACE2 and AXL, while the expression levels of the other three types of cells were comparable (Supplementary Figure S4C). Further, flow cytometry analysis revealed that the cell composition in the 3D cultured spheroids is comparable to that in SAEC differentiated under ALI condition, with 19.00 ± 2.92% ciliated cells, 3.40 ± 0.43% goblet cells, 14.51 ± 1.59% club cells, and 19.11 ± 2.38% basal cells (Supplementary Figure S4D). Furthermore, similar to SAEC differentiated under ALI condition, majority of all above of four types of cells express both ACE2 and AXL, and goblet cells express higher levels of ACE2 and AXL than other three cells types (Supplementary Figures S4E,F).

## Discussion

*In-vitro* differentiated human primary airway epithelial cells are a valuable tool for studying the interaction between SARS-CoV-2 and host cells. However, to generate a physiologically relevant *in-vitro* modeling system, several factors must be considered, including epithelial cell type, cell donor, and cell culture conditions. In the present study, we examined the expression of SARS-CoV-2 entry genes, including *ACE2, TMPRSS2, CTSL*, and *AXL* in primary human airway epithelial cells isolated from 11 lung cancer patients. Our results show that (i) the expression of SARS-CoV-2 entry genes is comparable between SAEC and HBEC, except for higher expression of *AXL* in HBEC than in SAEC, (ii) ALI culture leads to higher expression of *ACE2, TMPRSS2* and *CTSL* in both HBEC and SAEC than submerged culture, (iii) a negligible association was found between the expression of SARS-CoV-2 entry genes and donor-related parameters such as age, smoking status, and disease co-morbidities, (iv) *in-vitro* differentiated SAEC, either differentiated under ALI conditions or differentiated in 3D cultures express comparable levels of ACE2 and AXL on ciliated cells, goblet cells, club cells, and basal cells.

Using HBEC and SAEC isolated from the same donors, we were able to compare the expression of SARS-CoV-2 entry genes in the two cell types. To our knowledge, this is the first report describing differences in SARS-CoV-2 entry gene expression between donor-matched two types of *in-vitro* differentiated primary airway epithelial cells. Unexpectedly, no significant difference was found between HBEC and SAEC with respect to the expression of *ACE2, TMPRSS2*, or *CTSL*. This finding suggests that both HBEC and SACE are suitable for the *in-vitro* modeling system for investigating SARS-CoV-2. Previously, Aguiar and colleagues compared the expression of SARS-CoV-2 entry genes, including *ACE2, TMPRSS2*, and *CTSL*, in airway epithelial cells collected from bronchial brushings of the trachea, large airways, and small airways (10). Their results show that the expression of *ACE2* is highest in the trachea and lowest in the small airways, the expression of *TMPRSS2* is not significantly different among the three groups, and the expression of *CTSL* is highest in the small airways and lowest in the trachea. There are two possible reasons for the discrepancy between our results and the results of Aguiar's study. First, we examined gene expression from *in-vitro* differentiated primary human airway epithelial cells, whereas Aguiar focused on samples from bronchial brushings. The *in-vitro* differentiation process could alter the expression profile of SARS-CoV-2 entry genes. In contrast to our study, in which HBEC and SAEC were from the same donors, in Aguiar's study bronchial brushes were collected from different parts of the airways from different donors.

In addition to *ACE2*, it was recently suggested by Wang et al. (4) that *AXL* is another entry receptor for SARS-CoV-2. In their study, Wang et al. show that the expression of *ACE2* in lung and epithelial cells is much lower than that of *AXL*, suggesting that virus entry mediated by *AXL* plays an important role in promoting SARS-CoV-2 infection. Our observations on *in-vitro* differentiated primary SAEC confirmed that the expression of *AXL* at the mRNA level is indeed much higher than that of *ACE2*. Furthermore, the current study shows that *in-vitro* differentiated HBEC express more *AXL* than SAEC under ALI conditions, whereas such difference was not observed between the two cell types in submerged cultures. This finding suggests that the two types of primary epithelial cells show different expression of *AXL* under different *in-vitro* cell differentiation status.

In particular, the expression of *ACE2, TMPRSS2*, and *CTSL* is significantly increased in cells differentiated under ALI conditions compared with submerged culture, and this effect is observed in both HBEC and SAEC. Compared with submerged culture, ALI conditions correspond much more closely to the physiological situation of the lung epithelium *in-vivo*, so that the biological effects observed here appear to be more relevant than those observed under the former conditions (17). For example, primary airway epithelial cells differentiated under ALI conditions are able to reproduce the transcriptional profile of human airway epithelium (18) and recapitulate *in-vivo* responses to cigarette smoke (19). Because ACE2, TMPRSS2, and CTSL are all involved in ACE2-mediated SARS-CoV-2 entry (20), the current study suggests that the expression of genes involved in ACE2-mediated virus entry is related to the state of cell differentiation and that *in-vivo*-like differentiation conditions increase the expression of these genes. Based on these results, it is conceivable that primary airway epithelial cells differentiated under ALI conditions are better *in-vitro* modeling systems than submerged cultures for studying SARS-CoV-2 infection. Of note, while this paper was under review, a study by Guo et al. (12) has been published that clearly shows (i) ACE2 and TMPRSS2 expression is significantly increased in bronchial epithelial cells differentiated under ALI conditions compared to submerged cultured cells, (ii) differentiated ALI cultures are a suitable model to investigate SARS-CoV-2 infection in the presence or absence of therapeutic drugs. Therefore, findings from both the current study and Guo's study suggest that cells differentiated under ALI conditions are physiologically relevant models for investigating SARS-CoV-2 infection.

The expression of SARS-CoV-2 entry genes, particularly *ACE2*, in human airway epithelium has been shown to be related to demographic and clinical characteristics. For example, a recent study reported that increased gene expression of *ACE2* was associated with male sex (16), while Watson et al. (11) reported that *ACE2* expression did not correlate with age and was not different dependent on gender. It has been suggested that COPD and asthma, two pathogenic respiratory diseases, are associated with increased and decreased expression of *ACE2*, respectively (9, 15, 16). In addition, current smokers have been shown to be associated with increased expression of *ACE2* compared with ex-smokers and never smokers (9, 15, 21). Unexpectedly, our results show that the above clinical and demographic characteristics are not associated with the expression of *ACE2* and *AXL* in *in-vitro* differentiated SAEC. This discrepancy suggests that the effects of these characteristics on the expression of SARS-CoV-2 entry genes *in-vivo* are not transmitted during *in-vitro* cell differentiation in this modeling system. Therefore, the differences between *in-vitro* and patient sampled cells may be of note to *in-vitro* investigations in the future. The current study has two major limitations. First, although tumor-free, all airway epithelial cells used in this study were isolated from patients with lung cancer, which could have an impact on the results. Second, the number of samples used in this study is relatively small. Only 11 patients were recruited for the study, which could affect the results. For example, correlation analysis showed an inverse but non-significant correlation between age and expression of *TMPRSS2*. In addition, a tendency for higher expression of *AXL* was observed in SAEC from COPD patients than in the other two groups. It is not clear whether the lack of statistical significance was due to the lack of effect or the limitation due to the small sample size.

In summary, the present study demonstrates that *in-vitro* model selection, but not cell donor characteristics, is the main factor affecting SARS-CoV-2 entry gene expression in primary airway epithelial cells differentiated *in-vitro*. This finding suggests that culture conditions drowns out the patient characteristics in the regulation of expression of SARS-CoV-2 entry genes. Therefore, airway epithelial cells differentiated under ALI conditions are superior to those grown submerged as *in-vitro* model systems for studying SARS-CoV-2 infection.

## Data availability statement

The original contributions presented in the study are included in the article/Supplementary material, further inquiries can be directed to the corresponding author/s.

## Ethics statement

The studies involving human participants were reviewed and approved by Local Ethics Committee of the University Lübeck. Written informed consent for participation was not required for this study in accordance with the national legislation and the institutional requirements.

## Author contributions

BK, XYu, and FP were involved in the conception and design of the study. BK and XYue performed the experimental work. BK analyzed the data and generated figures. TG, AG, and CK contributed by providing essential materials (Lung biopsy and clinical information). XYu and BK were involved in drafting the manuscript. All authors were involved in revising the manuscript. All authors contributed to the article and approved the submitted version.

## Funding

This study was supported by the German Federal Ministry of Education and Research (BMBF) *via* German Center for Lung Research (DZL) and Airway Research Center North (ARCN).

## Conflict of interest

The authors declare that the research was conducted in the absence of any commercial or financial relationships that could be construed as a potential conflict of interest.

## Publisher's note

All claims expressed in this article are solely those of the authors and do not necessarily represent those of their affiliated organizations, or those of the publisher, the editors and the reviewers. Any product that may be evaluated in this article, or claim that may be made by its manufacturer, is not guaranteed or endorsed by the publisher.

## Abbreviations

ACE2: angiotensin converting enzyme 2
ALI: air-liquid interface
AXL: tyrosine protein kinase receptor UFO
CTSL: cathepsin L
COPD: chronic obstructive pulmonary disease
HBEC: human bronchial epithelial cells
SAEC: small airway epithelial cells
SM: submerged
TMPRSS2: transmembrane serine protease 2.
