# Supplementary material for: Air exposure and cell differentiation are essential for investigation of SARS-CoV-2 entry genes in human primary airway epithelial cells in vitro
Source: Front Med (Lausanne). 2022 Sep 6;9:897695. doi: 10.3389/fmed.2022.897695 (PMC9487839; doi:10.3389/fmed.2022.897695)
Supplement: Supplementary file 1 [file Data_Sheet_1.docx]

**Additional file 1**

**Table S1: Demographic and clinical features of individual patients**

| **Patient ID** | **Gender** | **Age (years)** | **Smoking status** | **Diagnosis of lung cancer** | **Stage** | **COPD/ asthma** | **Isolated cells** |
| --- | --- | --- | --- | --- | --- | --- | --- |
| 1 | male | 68 | Ex-smoker | ADC | IB (N0, M0) | --- | SAEC |
| 2 | male | 83 | Ex-smoker | SQCC | IIB (N1, M0) | --- | SAEC, HBEC |
| 3 | female | 69 | Smoker | ADC | IB (N0, M0) | --- | SAEC, HBEC |
| 4 | female | 75 | Ex-smoker | ADC | IB (N0, M0) | COPD | SAEC |
| 5 | male | 76 | Ex-smoker | ADC | IIA (N0, M0) | --- | SAEC, HBEC |
| 6 | male | 61 | Smoker | SQCC | IA (N0, M0) | Asthma | SAEC |
| 7 | male | 78 | Ex-smoker | ADC | IA2 (N0, M0) | Asthma | SAEC |
| 8 | male | 70 | Ex-smoker | ADC | IA2 (N0, M0) | COPD | SAEC |
| 9 | female | 57 | Smoker | NSCLC | IA (N0, M0) | Asthma | SAEC |
| 10 | female | 81 | Ex-smoker | ADC | IA2 (N0, M0) | Asthma | SAEC |
| 11 | male | 70 | Ex-smoker | ADC | IB (N0, M0) | COPD | SAEC, HBEC |

Squamous cell carcinoma, SQCC; non-small cell lung cancer, NSCLC; adenocarcinoma, ADC; chronic obstructive pulmonary disease, COPD; small airway epithelial cells, SAEC; human bronchial epithelial cells, HBEC; no lymph node involvement, N0; local lymph node involvement, N1; no metastases, M0.


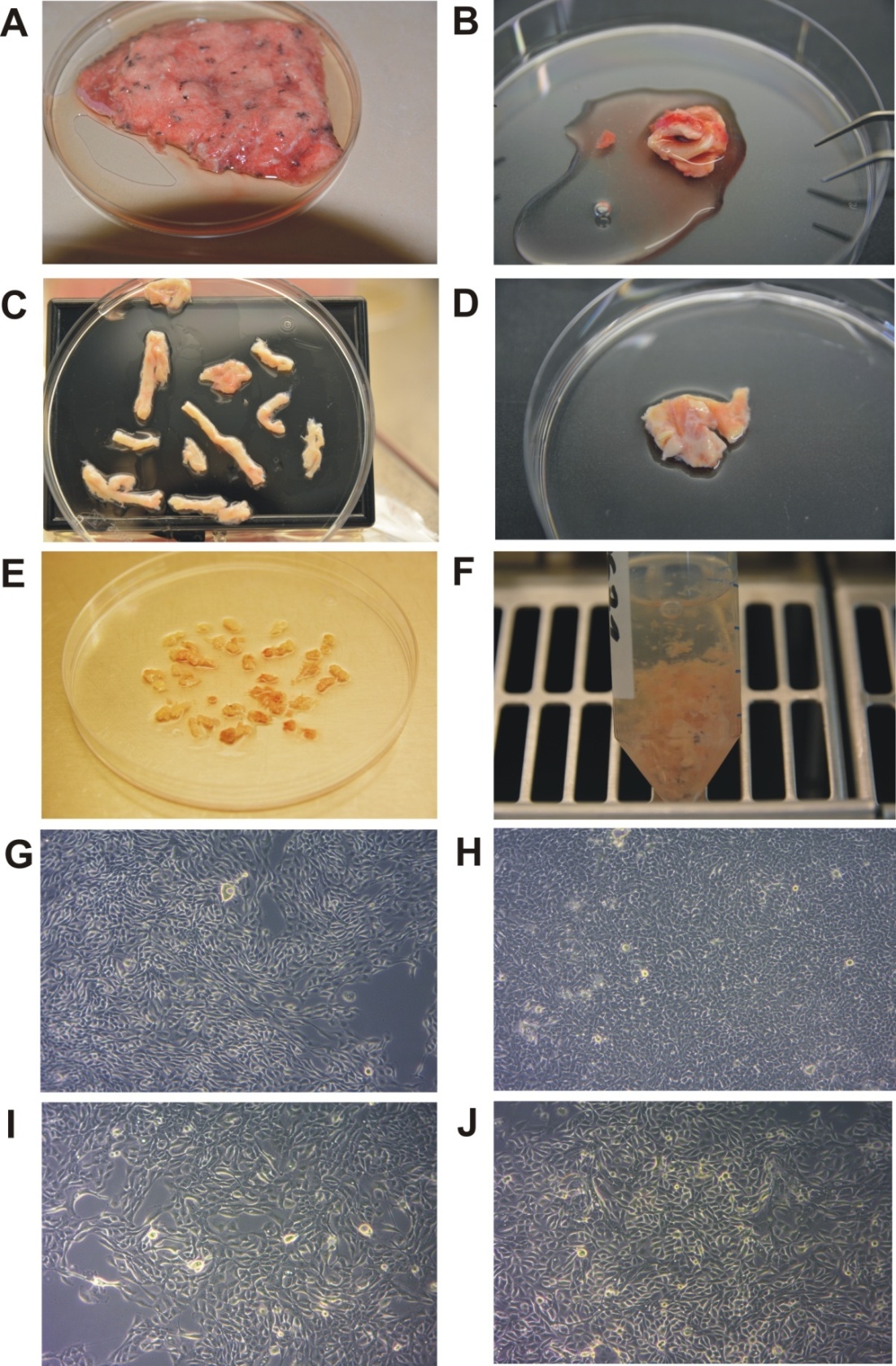


**Figure S1. Isolation of airway epithelial cells.** Human lung tissue (A) or bronchus (B) were used as starting material. (C and D) Excess surrounding tissue was removed. Subsequently, tissue was cut into smaller pieces (E) and placed into dissociation buffer (F) to separate cells. (G-J) Isolated cells were grown on collagen I-coated petri dishes in expansion medium. (G) Cells in passage 1, (H) passage 4, (I) passage 7, (J) passage 11.


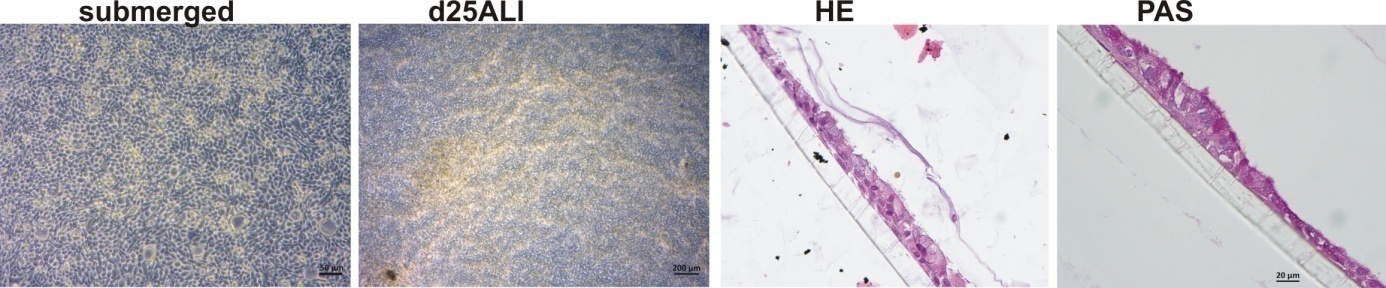


**Figure S2.** Morphology of cells grown in submerged/ALI culture. Using immunohistochemistry mucus was visualized by Periodic acid–Schiff (PAS) stain and ciliated cells can be seen in Hematoxilin-Eosin (HE) as well as PAS stain.


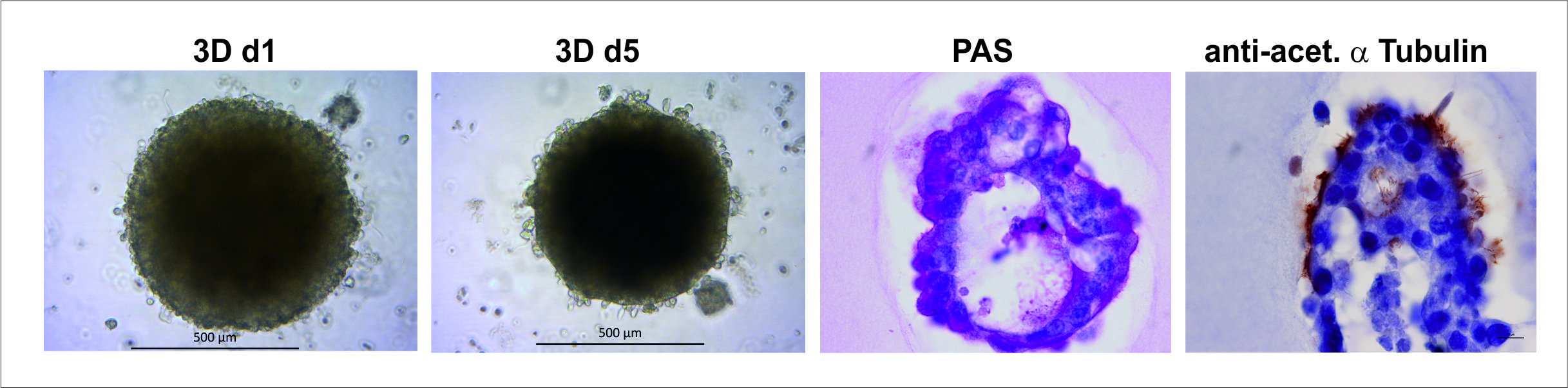


**Figure S3. Surface expression of ACE2 and AXL on SAEC differentiated in 3D culture.** SAEC were differentiated under 3D conditions, and spheroids were analyzed for the presence of goblet cells by Periodic acid–Schiff (PAS) stain and ciliated cells by detecting acetylated α- Tubulin using immunohistochemistry.


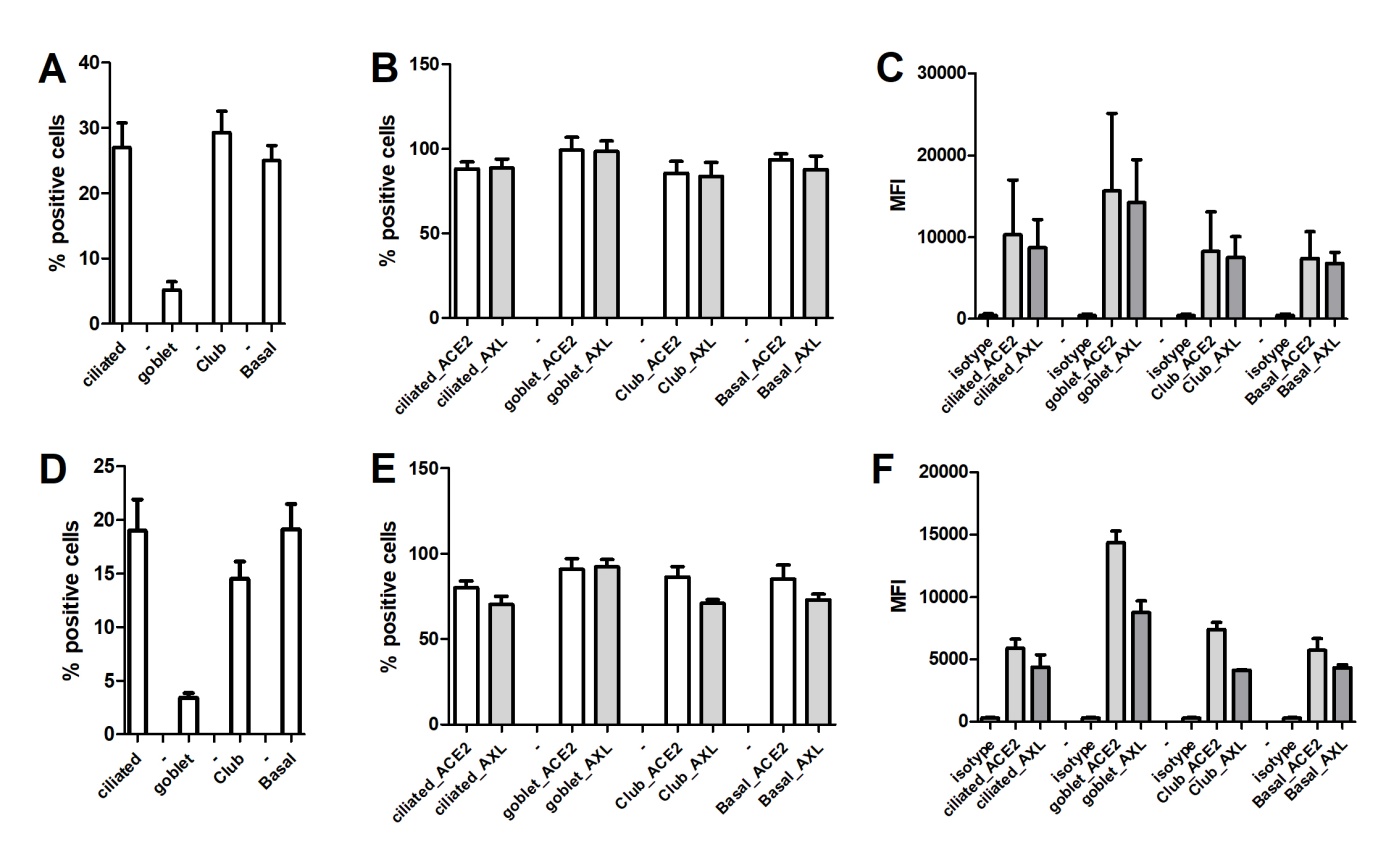


**Figure S4: Surface expression of ACE2 and AXL on differentiated SAEC.** (A-C) SAEC were differentiated under ALI conditions (4 patients, 5 cultures), or (D-F) SAEC were cultured under 3D conditions (2 patients, 3 cultures). For flow cytometric analysis cells were detached from the transwell membrane (ALI) or were disaggregated by careful resuspension (3D). After staining the cells were analyzed for cellular composition (A/D), frequencies of ACE2 or AXL positive cells (B/E), and expression level of ACE2 or AXL indicated by mean fluorescence intensity (MFI; (C/F)).
